# A New Styptic Collodion

**Published:** 1869-11

**Authors:** 


A New Styptic Collodion.—M. Carlo Paresi gives, in the *Gazette de Turin*, the following recipe:—Collodion 100 parts, carbolic acid 10 parts, tannin 5 parts, benzoic acid 3 parts. Agitate until a perfect solution is formed. It is of a brownish color, gives a pellicle similar to ordinary collodion and instantly coagulates blood.—*Indian Medical Gazette*, July 1, 1869.

